# Genome Evolution in Three Species of Cactophilic *Drosophila*

**DOI:** 10.1534/g3.116.033779

**Published:** 2016-08-03

**Authors:** Alejandro Sanchez-Flores, Fernando Peñaloza, Javier Carpinteyro-Ponce, Nestor Nazario-Yepiz, Cei Abreu-Goodger, Carlos A. Machado, Therese Ann Markow

**Affiliations:** * Unidad de Secuenciación Masiva y Bioinformática, Instituto de Biotecnología, Universidad Nacional Autónoma de México (UNAM), Cuernavaca, Morelos, 62210 Mexico; † Laboratorio Nacional de la Genomica Para la Biodiversidad, CINVESTAV, Irapuato, Guanajuato, 36821 Mexico; ‡ Department of Biology, University of Maryland, College Park, Maryland 20742; § Division of Biological Sciences, University of California at San Diego, La Jolla, California 92093

**Keywords:** *Drosophila*, evolution, genome, inversions, cactophilic

## Abstract

We report genomes of two species of cactophilic *Drosophila*: *Drosophila arizonae* and *D. navojoa*. These two are the closest relatives of *D. mojavensis*, forming the *D. mojavensis* cluster. *D. mojavensis* and *D. arizonae* diverged from *D. navojoa* ∼5.8 Mya, while the split between *D. arizonae* and *D. mojavensis* is more recent, at 1.5 Mya. Together the three genomes provide opportunities to examine genomic changes associated with speciation and host shifts in this ecologically defined group of flies. The three species are also separated by fixed inversion differences in three of their six chromosomes. While the levels of nucleotide divergence in the colinear chromosomes are significantly lower than in the inverted chromosomes, consistent with a past role of the inversions in preventing gene flow, the patterns differ among the inverted chromosomes when the locations of nucleotides inside or outside of the inversions are considered. For Muller element E, there is greater divergence external to the inversion breakpoints. For Muller A, the divergence is slightly higher inside the inversions, while for Muller B, the breakpoints and hence the difference in substitutions in relation to the inversions could not be determined. The differences among the inverted chromosomes, especially once the breakpoints are clearly established, could aid in dating the origins of the inversions.

Historically, *Drosophila* species have been popular models for studies of evolution. The comparative analysis of the genomes of 12 *Drosophila* species in 2007 (Drosophila 12 Genomes Consortium 2007) continues to generate new insights into evolutionary processes at multiple time scales. Over 2000 species in the genus have radiated into a wide array of ecological niches, including decaying fruits, vegetables, flowers, mushrooms, slime fluxes, cacti, and soil (Markow and O’Grady 2005a, 2008). This striking variation in resource specialization is rivaled only by the diversity in their behavior and reproductive biology (Markow and O’Grady 2005b). Because well-defined phylogenetic relationships reveal recently evolved species, we can select species to investigate the earliest events and processes in evolution. Recent sequencing of the genomes of multiple and related *Drosophila* species of the *Drosophila melanogaster* group, for example, provides a far more complete picture of speciation than is available from studies of specific genes (Garrigan *et al.* 2012).

One of the best-characterized *Drosophila* radiations is the group of flies that utilize necrotic cactus as a breeding site (Heed 1978, 1982). The large *D. repleta* species group contains at least 100 species that breed in cactus in North and South America (Markow and O’Grady 2005a). Some host shifts have occurred between closely related and thus chemically similar cacti, while other shifts have been between very different types of cacti. In addition to the ecological shifts that accompanied the multiple speciation events are the reproductive isolating mechanisms that span premating, postmating-prezygotic, and postzygotic incompatibilities. Finally, many of the closely related species in the *D. repleta* group have fixed chromosomal rearrangements, which no doubt underlie much of their genomic and phenotypic divergence (Kirkpatrick and Barton 2006). Despite their cactophilic lifestyles, many *D. repleta* group species are relatively easy to maintain in the laboratory and thus useful for manipulative experimentation.

Owing to its ecology and position in the subgenus *Drosophila*, *D. mojavensis* (Markow and O’Grady 2005a; Heed 1978, 1982), was among the first non-*D. melanogaster* species to have a fully sequenced genome (Drosophila 12 Genomes Consortium 2007). *D. mojavensis* is part of a triad of species that also includes its sister species, *D. arizonae*, and *D. navojoa* (Ruiz *et al.* 1990), known as the *D*. *mojavensis* cluster (Figure 1). Despite their close evolutionary relationship, the three species exhibit a number of distinct ecological and evolutionary differences, the basis of which can be addressed with genomic data. Basal among the three is *D. navojoa*, an *Opuntia* breeder whose distribution is restricted to the west coast of Mexico’s mainland. *D. mojavensis* is more widespread, occurring in southern California, Arizona, Sonora, Sinaloa, and the Baja California peninsula. The most widespread is *D. arizonae*, with populations reported from Guatemala, throughout Mexico, and to California and Arizona. The ability to utilize columnar cacti as hosts appears to have occurred with the evolution of *D. mojavensis* and *D. arizonae*, although both species utilize *Opuntia* on parts of their ranges.

**Figure 1:**
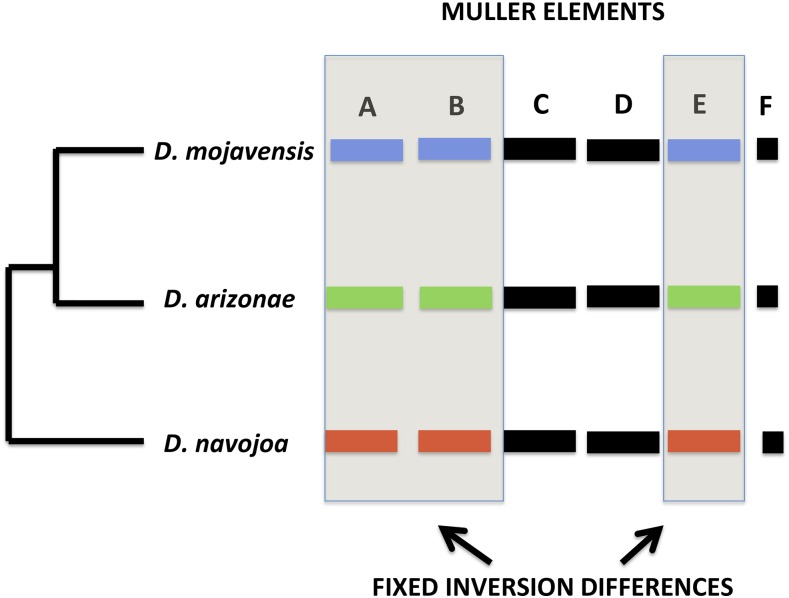
Fixed Inversion Differences among the three species in the *Drosophila mojavensis* cluster and indication of Muller elements that are colinear (C, D, and F) or that have fixed chromosomal inversions between species (after Wasserman 1962, 1992). Colinear chromosomes are in black, while red, blue, and green are fixed inversion differences among the three species.

Furthermore, the three also are “good” species in nature, in that although adults may be collected from the same host cacti in areas of sympatry, hybrids have never been found in the wild (Ruiz and Heed 1988). All three species can be crossed in the laboratory but with varying degrees of success because of behavioral isolation, postmating-prezygotic incompatibilities, and hybrid sterility and/or inviability (Ruiz *et al.* 1990; Markow 1982; Hardy *et al.* 2011).

All three species have the same six chromosomes, each of which corresponds to one of the six *Drosophila* Muller elements. Three of the chromosomes are colinear, *i.e.*, have the same gene order (Wasserman 1962, 1992; Ruiz *et al.* 1990), while the other three each contain inversion differences that are fixed among the species (Figure 1). An earlier, low-resolution study using 10 sequence markers found no evidence of introgression between *D. mojavensis* and *D. arizonae*, but did not rule out genetic exchange in the colinear chromosomes in the past based on patterns of sequence divergence in chromosomes harboring fixed inversions *vs.* chromosomes that are colinear (Machado *et al.* 2007a). Recent work using genome-wide short read data supports that previous finding (Lohse *et al.* 2015). The increased accessibility of whole genome sequencing allows us to examine in greater depth the role of the chromosome inversions in divergence between all three species. Nucleotide divergence among the three species should differ depending upon whether the inverted or colinear chromosomes are examined and, given that *D. navojoa* is an outgroup to the other two and is restricted to *Opuntia*, the greatest divergence should be between this species and the two derived ones. Here, we report the newly sequenced genomes of *D. arizonae* and *D. navojoa* and examine the evolutionary differences in coding regions between species for colinear and inverted chromosomes.

## Materials and Methods

### Genome sequencing

We performed whole genome sequencing on DNA extracted from adult males of inbred lines of *D. arizonae* and *D. navojoa* using paired-end and Nextera mate pair libraries (3KB insert size) that were constructed and sequenced at the Hudson Alpha Institute using Illumina HSeq2000. The *D. arizonae* originally was from an isofemale strain collected near Guaymas Sonora and the *D. navojoa* was a strain collected in Jalisco, México. Each strain has been deposited in the UCSD Drosophila Species Stock Center.

### Genome assembly and gene prediction

Data preparation and *de novo* genome assembly were performed using AllPaths-LG (R48777) software (Gnerre *et al.* 2011), including both paired-end and mate pair libraries. The parameters PLOIDY = 2 and GENOME_SIZE = 150000000 were specified to the data preparation module (PrepareAllPathsInputs.pl) for both genomes. For the assembly pipeline (RunAllPathsLG), default parameters were used. Each assembly was improved with GapFiller v1.1 (Boetzer and Pirovano 2012) to remove gaps, and five iterations of ICORN2 (Otto *et al.* 2010) and REAPR (Hunt *et al.* 2013) were conducted to close gaps between contigs, correct base errors, and break misassembled regions, respectively.

The completeness assessment for each genome assembly was performed using the Core Eukaryotic Genes Mapping Approach (CEGMA) v2.5 pipeline (Parra *et al.* 2009) using the 248 Core Eukaryotic Genes (CEGs) models from CEGMA for each species. The gene prediction software AUGUSTUS v3.0.3 was trained for gene prediction using the protocol found here: http://www.molecularevolution.org/molevolfiles/exercises/augustus/training.html. Genome assemblies have been deposited in GenBank under accession numbers LSRL00000000 (*D. navojoa*) and LSRM00000000 (*D. arizonae*).

### Functional annotation

The protein products from the conceptual translation for each gene model in both species were annotated by comparing against protein sequences from *Drosophila* species downloaded from FlyBase.org (release February 2014), and clustered using CD-HIT v.4.6 (Li and Godzik 2006), with a cutoff value of 80% identity using default parameters. The Uniprot ID and short name for each protein was obtained by matching the FlyBase IDs in the clusters with custom Perl scripts and relational files obtained from Uniprot (ftp://ftp.uniprot.org/pub/databases/uniprot/current_release/knowledgebase/complete/docs/fly.txt and ftp://ftp.uniprot.org/pub/databases/uniprot/current_release/knowledgebase/complete/docs/shortdes.txt).

### Comparative genomics analyses

Genome alignments were performed using ABACAS (Assefa *et al.* 2009) against the *D. mojavensis* Muller elements. As an additional quality check, paired-end reads were mapped over the Muller elements using SMALT v.7.4 (ftp://ftp.sanger.ac.uk/pub/resources/software/smalt/). The paired-end and mate pair libraries were analyzed independently.

### Coding sequence analyses

A total of 5952 single copy orthologous sequences from predicted genes for *D. arizonae*, *D. navojoa*, and the published genome of *D. mojavensis* were assigned using OrthoMCL (Li *et al.* 2003). To avoid potential annotation artifacts, such as false paralogs and miss-annotations, only single copy ortholog gene clusters present in all three species represented were included. Orthologous gene clusters were aligned using PRANK v1.4 (Löytynoja and Goldman 2005). All uninformative sites for each alignment were removed using Gblocks 0.91b (Castresana 2000). Pairwise nonsynonymous (dN) and synonymous (dS) substitutions and dN/dS ratios among the three species were estimated with the yn00 algorithm implemented in PAML package v4.8 (Yang 2007). Given that very small values of dS can lead to high values of dN/dS (Wolf *et al.*, 2009), only orthologs with dN/dS < 2 were included in the comparisons for each species pair.

Using the *D. mojavensis* genome as a reference, orthologous genes were divided and clustered according to their chromosome position in each Muller element, to examine differences between inverted and colinear chromosomes. Chromosomes X, 2, and 3 have fixed rearrangements between *D. mojavensis* and both *D. arizonae* and *D. navojoa* (inversions Xe, 2q-2r-2s, and 3d), and were classified as “inverted” (Wasserman 1962; Ruiz *et al.* 1990). Chromosomes 4 and 5 do not have fixed rearrangements between the species and were classified as “colinear.” Genes from the colinear small dot chromosome (Muller F) were not included in the analyses. Because the breakpoints of the fixed inversions in *D. mojavensis* are known for chromosomes X (Muller element A, inversion Xe) and 2 (Muller element E, inversions 2q-2r-2s) (Runcie and Noor 2007; Guillén and Ruiz 2012), differences between the genes localized inside the inversion and those outside were determined for each of these chromosomes. Given that chromosome 2 has three overlapping inversions that differentiate *D. mojavensis* (inversions 2q-2r-2s), the largest inverted region defined by the proximal and distal breakpoints from inversions 2q and 2r were used to define the inverted region in the analyses (Guillén and Ruiz 2012). Colinear and inverted regions were defined as those regions flanking the inversion breakpoints and those in between the inversion breakpoints, respectively (*i.e.*, the regions outside or inside the inversion loops in an inversion heterozygote). The nonparametric Mann–Whitney *U*-test was employed to determine the significance level for the differences between the inverted and colinear regions.

### Positive selection analyses

Branch-site analyses (model = 2 and nsites = 2) implemented in PAML package v4.8 (Yang 2007) were performed to detect evidence of positive selection allowing sites with ω > 1 among lineages *D. arizonae*, *D. navojoa*, and *D. mojavensis*. Orthologous genes clustered by Muller element were analyzed by testing for positive selection in each branch of the phylogeny (foreground branches) separately. Likelihood ratio tests at *P* < 0.05 were performed to reject the null model of ω = 1 in the foreground branches.

### Gene ontology enrichment analysis

To determine the overrepresented functional categories for the genes putatively under positive selection in each lineage, gene ontology (GO) enrichment analyses were performed using the Database for Annotation, Visualization and Integrated Discovery (DAVID) resource v6.7 (Huang *et al.*, 2009). For each *D. mojavensis* gene, GO terms were transferred from their orthologous sequence in *D*. *melanogaster*. Only genes with a *D. melanogaster* ortholog and an associated GO term were included in the analysis.

### Divergence time estimation

Divergence times for *D. arizonae*, *D. navojoa*, and *D. mojavensis* were estimated using a set of 5704 single copy orthologous genes shared among the three species and the outgroup *D. virilis*. All orthologous gene clusters were aligned and noninformative sites were removed using PRANK v1.4 (Löytynoja and Goldman 2005) and Gblocks (Castresana 2000), respectively. All gene alignments were concatenated for each species, in order to obtain a single alignment for the three species. Divergence time estimation was performed using BEAST2 (Bouckaert *et al.*, 2014), assuming a relaxed-clock model of evolution among branches modeled by a log-normal distribution. Sequence evolution was modeled using a symmetrical model (Zharkikh 1994) with γ distributed rate variation among sites estimated by jModeltest v2.1.7 (Darriba *et al.*, 2012). We assumed a Yule process (Heled and Drummond 2012) as a model for prior speciation.

The time calibration used for divergence on *D. virilis* and the *D. repleta* group assumed a normal distribution with a mean of 32 Mya and Σ = 0.3, as estimated by Russo *et al.* (1995), and more recently by Obbard *et al.* (2012). Node dates for *D. navojoa*, *D. mojavensis*, and *D. arizonae* were then estimated using the prior default parameters in BEAST2. Markov chain Monte Carlo (MCMC) chain length was set to 10^8^ steps in order to reach a sufficient effective sample size. Runs were performed twice to confirm stationary *a posteriori* distributions.

### Data availability

The authors state that all data necessary for confirming the conclusions presented in the article are represented fully within the article.

## Results

### Sequencing and assembly of the D. arizonae and D. navojoa genomes

Sequencing and assembly results for *D. arizonae* and *D. navojoa* are summarized in Table 1. The estimated genome size for *D. arizonae* was 142.06 Mb with 42% GC, while *D. navojoa* has a 148.68 Mb genome with 39% GC. The repetitiveness, based on a Kmer (K = 25) analysis of the reads for each genome, was 10% for *D. arizonae* and 17% for *D. navojoa*. Assembly completeness values are shown in Table 1; the *D. arizonae* genome assembly is the most complete genome with 93.95% and 97.58% values for the CEG complete and partial models, respectively. In the case of *D. navojoa*, the result was a more fragmented assembly with complete/partial CEGMA values of 78.23% / 84.68%. Despite the fragmentation level of each assembly, the scaffolds were aligned and ordered using the six Muller elements from *D. mojavensis* and the coverage statistic is described for each species in Table 1.

**Table 1:** Sequencing statistics and quality control, and assembly statistics

	*D. arizonae*	*D. navojoa*
Sequencing statistics and quality control		
Read length (bases)	2 × 100	2 × 100
PE reads	97,359,954	158,820,658
PE insert size (bases)	145 ± 60	257 ± 60
MP reads	37,994,866	42,353,652
MP insert size (bases)	1717 ± 796	2345 ± 489
Estimated genome size (Mb)	142.06	148.68
Estimated genome coverage	52×	81×
GC content (%)	42	39
Repetitiveness (%)	10	17
Assembly statistics		
Bases in the assembly (Mb)	141.37	115.88
Bases in Muller elements (Mb)	132.58	95.27
Total no. of scaffolds	3179	8054
Average scaffold size (kb)	44.47	14.39
Shortest scaffold length (bases)	886	867
N50 (Mbases / no. scaffolds)*^a^*	2.65 / 3	2.18 / 3
N90 (kbases / no. scaffolds)*^a^*	71.37 / 7	4.31 / 929
CEGMA complete / partial (%)	92.42 / 97.58	78.23 / 84.68
Muller element coverage (%)	92.35	82.21

a Statistic including scaffolds ordered and linked into Muller elements.

### Gene prediction and annotation

For *D. arizonae* and *D. navojoa*, the gene prediction process was performed by training AUGUSTUS with the 248 CEG protein products obtained from CEGMA. Table 2 summarizes the statistics for the gene prediction and conceptual translations for all three genomes. In general, gene structure and protein length were similar for the three species, with only slight differences.

**Table 2:** Protein-coding gene models and amino acid sequence statistics

	*D. arizonae*	*D. navojoa*	*D. mojavensis*
Predicted protein-coding genes	12,129	10,695	15,015
Average gene length (bases)	2176.23	2029.55	2138.78
Average protein length (aa)	724.409	675.517	711.928
Average transcript length (bases)	2638.08	2499.65	2677.92
Average exon length (bases)	385.915	371.348	374.89
Average exon number per gene	5.63913	5.46536	5.70509

### Comparative genomics of the D. repleta group

The contigs from each assembly were ordered against the six *D. mojavensis* Muller elements using the program ABACAS (Assefa *et al.* 2009) to obtain a pseudomolecule that was used for further comparative genomics analysis. As a quality check, we mapped all the raw reads to the *D. mojavensis* Muller elements to calculate the coverage per Muller element. Further, we corroborated the location of the breakpoints for the fixed inverted regions in chromosomes X (Muller A) and 2 (Muller E) of *D. mojavensis*, looking for aberrant distances between mate pair reads from *D. arizonae* and *D. navojoa* that mapped to proposed breakpoint locations (Runcie and Noor 2007; Guillén and Ruiz 2012). The location of the breakpoints for inversion Xe in Muller A, fixed in *D. mojavensis*, were supported in *D. arizonae* by 31 and 112 mate pair reads (for each breakpoint) with aberrant insert sizes, and in *D. navojoa* by 176 and 494 mate pair reads (for each breakpoint) with aberrant insert sizes that matched the size of the inverted region (∼10 Mb). For Muller element E we found similar results for both species that matched previously mapped locations of the fixed inverted region in *D. mojavensis* (Guillén and Ruiz 2012).

### Sequence divergence in inverted and colinear chromosomes

**Figure 2:**
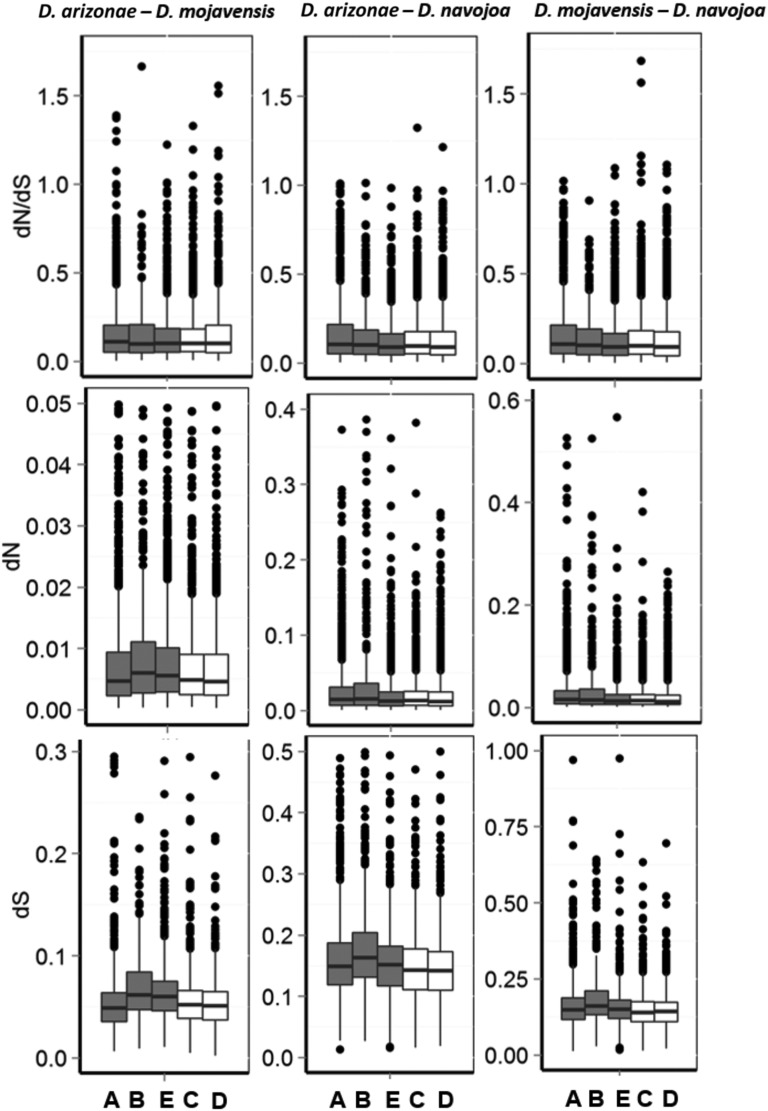
Distribution of nonsynonymous (dN) and synonymous (dS) substitution rates per site and dN/dS ratios for ortholog genes in each Muller element among pairwise comparisons of *Drosophila mojavensis*, *D. arizonae*, and *D. navojoa*. Muller elements A, B, and E (gray bars) have fixed inversion differences among species. Muller elements C and D (white bars) are colinear in the three species. The number of genes used in each comparison are shown in Table S4.

**Figure 3:**
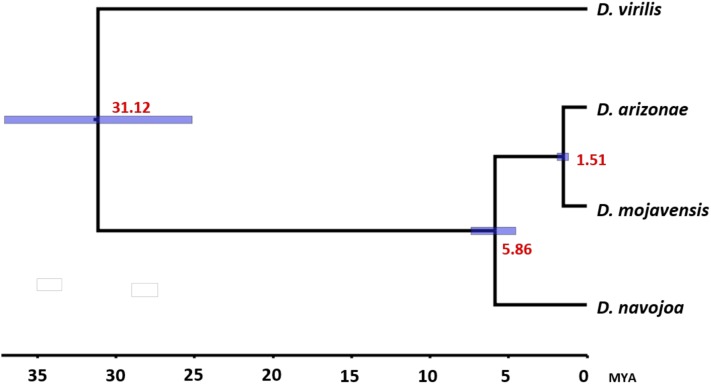
Divergence times within the *D. repleta* group with *D. virilis* as an external group. The node dates (in red) were estimated under an uncorrelated log-normal relaxed clock. The 95% highest posterior density intervals (in blue) are shown for each node.

Coding sequences from chromosomes that have fixed inversion differences between *D. mojavensis* and *D. arizonae* show significantly higher levels of nucleotide divergence than coding sequences from colinear chromosomes (Supplemental Material, Table S1), although the results also hold for the comparisons with the outgroup *D. navojoa*. As expected, the *D. mojavensis* and *D. arizonae* comparisons have lower average divergences than the other two pairwise comparisons that included *D. navojoa* (Mann–Whitney *U*-test; *P* < 0.001), consistent with the phylogenetic relationships of the three species. When analyzing each chromosome separately, we still find significant differences among each inverted chromosome and the combined noninverted chromosomes (Table 3 and Figure 3). Muller E shows significantly higher dS and dN only in the *D. mojavensis–D. arizonae* comparison, consistent with the idea that the inverted region in that chromosome played a role in reducing introgression during species divergence. Muller A and B, however, show significantly higher dS and dN not only in the *D. mojavensis–D. arizonae* comparison, but also in the comparisons that include the outgroup *D. navojoa* (Table 3 and Figure 2). For those two chromosomes, the connection between the fixed inverted regions and reduced introgression during the *D. mojavensis–D. arizonae* divergence is less clear, as the pattern of increased divergence relative to the colinear chromosomes could be the result of an overall increased substitution rate in the two inverted chromosomes.

**Table 3:** Average synonymous (dS) and nonsynonymous (dN) substitutions per site and dN/dS ratios for genes located in each Muller element in *D. arizonae* (ar), *D. mojavensis* (mo), and *D. navojoa* (na)

	dN			dS			dN/dS		
Element	ar-mo	ar-na	mo-na	ar-mo	ar-na	mo-na	ar-mo	ar-na	mo-na
Muller A*^a^*	0.010 ↑	0.030 ↑	0.031 ↑	0.055	0.162 ↑	0.165 ↑	0.165	0.162	0.164
Muller B*^a^*	0.016 ↑	0.038 ↑	0.035 ↑	0.080 ↑	0.198 ↑	0.199 ↑	0.161	0.150	0.147
Muller E*^a^*	0.011↑	0.022	0.023	0.069 ↑	0.158	0.158	0.147	0.128	0.129
Muller C	0.008	0.022	0.022	0.055	0.151	0.148	0.163	0.118	0.145
Muller D	0.009	0.022	0.023	0.058	0.151	0.152	0.159	0.139	0.141

↑ indicates chromosomes with significantly higher divergence levels (Mann–Whitney *U*-test; *P* < 0.05); comparisons were performed between each Muller element and the combined inverted or colinear chromosomes.

a Chromosomes with fixed inversion differences between *D. arizonae* and *D. mojavensis*.

To explore this issue in more detail, we used information about the location of the inversion breakpoints in *D. mojavensis* for Muller A (Runcie and Noor 2009) and Muller E (Guillén and Ruiz 2012) to compare differences between genes located inside and outside the fixed chromosomal inversions that separate *D. mojavensis* and *D. arizonae* (Table 4). For Muller E, we observed that the level of divergence at synonymous sites is significantly higher for genes inside the inversion than in the colinear region of the chromosome (*P* = 0.00028), as expected if the inversion contributed to reduced introgression in that genomic region. Interestingly, dS was significantly higher for genes outside the inversion in the comparisons with the *D. navojoa* outgroup (*P* < 0.001). For Muller A, we observed that for all three species pairwise comparisons there are no significant differences in divergence (dN or dS) between genes located inside or outside the mapped single inversion (Xe) in that chromosome. In the context of the divergence of *D. mojavensis* and *D. arizonae*, this result suggests that this is a fairly differentiated chromosome where introgression across the whole chromosome stopped early during the divergence process (see *Discussion*). The inversion breakpoints for Muller B have not been mapped. We note, however, that this chromosome has the largest number of putatively selected genes in *D. navojoa* (controlling for chromosome gene number, see below), which could be part of the explanation behind the high divergence levels observed in the comparisons that include this outgroup species.

**Table 4:** Average synonymous (dS) and nonsynonymous (dN) substitutions per site and dN/dS ratios for genes located inside (IR) or outside (CR) the fixed inverted region in Muller elements A and E in *D. arizonae* (ar), *D. mojavensis* (mo), and *D. navojoa* (na)

	dN			dS			dN/dS		
	ar-mo	ar-na	mo-na	ar-mo	ar-na	mo-na	ar-mo	ar-na	mo-na
Muller A									
Genes in IR	346	387	387	346	387	387	346	387	387
Genes in CR	666	760	759	666	760	759	666	760	759
Median for IR	0.005	0.016	0.016	0.049	0.149	0.149	0.108	0.110	0.118
Median for CR	0.004	0.014	0.014	0.048	0.149	0.148	0.109	0.104	0.108
* P*-value	0.234	0.382	0.268	0.094	0.679	0.966	0.720	0.379	0.308
Muller E									
Genes in IR	859	930	946	859	930	946	859	930	946
Genes in CR	277	310	312	277	310	312	277	310	312
Median for IR	0.005	0.012	0.012	0.0616	0.1487	0.148	0.098	0.09	0.092
Median for CR	0.005	0.013	0.014	0.0561	0.1628	0.1614	0.103	0.092	0.088
* P*-value	0.591	0.420	0.309	0.0002*^a^*	0.0001*^a^*	0.0005*^a^*	0.622	0.501	0.903

a Significantly different (*P* < 0.05).

Finally, when comparing *D. arizonae* and *D. mojavensis*, we observed that the divergence (dS and dN) of genes located in Muller B is significantly higher than Muller E and Muller A (Muller B > Muller E > Muller A) (Table 3). This result has some possible implications for understanding the relative time of origin of each set of fixed inversions during the divergence of *D. arizonae* and *D. mojavensis* (see *Discussion*).

### Divergence estimation within the D. repleta group

The divergence between the three species was calculated using a set of 5704 single copy orthologous genes from the OrthoMCL clusterization, with *D. virilis* as an external group. Figure 3 and Table S2 show the divergence times between the *D. repleta* group and *D. virilis*. According to our calculations, the *D. virilis* and *D. repleta* group diverged 31.13 Mya, a result consistent with previous studies (Table S2). Inside the *D. repleta* group, the separation between *D. navojoa* and the other two species was 5.86 Mya. Finally, we estimate that *D. mojavensis* and *D. arizonae* diverged 1.51 Mya. The relationship between the newly sequenced genomes and those reported by the Drosophila 12 Genomes Consortium (2007) is presented in Figure S1.

### Genes under positive selection

**Figure 4:**
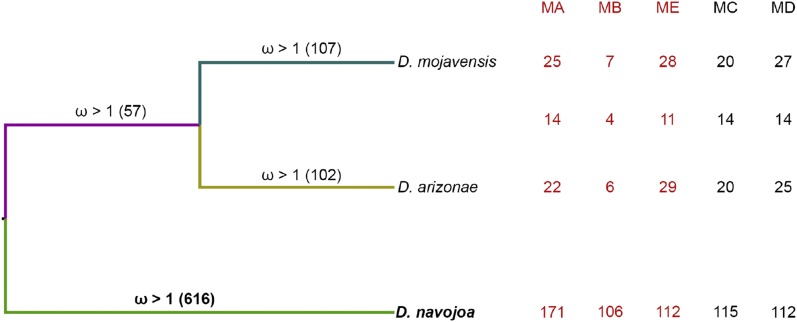
The number of putatively selected genes in each lineage is shown on each branch. The table on the right shows the numbers separated by Muller element, highlighting in red the chromosomes with fixed inversion differences between *D. mojavensis* and *D. arizonae*.

We used the topology from Figure 2 to conduct branch-site analyses (Yang 2007) to detect evidence of positive selection in each phylogenetic lineage (*D. arizonae*, *D. mojavensis*, and *D. navojoa*, ancestor of *D. arizonae–D. mojavensis*) (Figure 4). Interestingly, there are no significant differences in the number of genes under positive selection between *D. arizonae* and *D. mojavensis* lineages (*P* = 0.7801), but a very significant increase in the *D. navojoa* lineage (*vs.* *D. mojavensis*: *P* = 1.2 e-84; *vs. D. arizonae*: *P* = 8.2 e-87). Controlling for chromosome gene number, there are no significant differences in the number of positively selected genes among chromosomes with fixed inversions (Muller A, B, and E) in *D. mojavensis* or *D. arizonae*. However, there are significant differences among chromosomes in the number of positively selected genes in *D. navojoa*, with the highest numbers in Muller B (*P* < 0.0001 *vs.* Muller A and E), followed by Muller A (*P* < 0.0001 *vs.* Muller E).

GO term analyses show enrichment of few biological processes related to catabolic functions in *D. arizonae* (Table S3). In *D. mojavensis* there is an enrichment of genes involved in transcription regulation, and multiple metabolic processes that may be linked to host specialization (Table S3). Forty-eight diverse biological process genes show enrichment among the selected genes in the *D. navojoa* lineage (Table S3), a result that may be connected to the specialization of these flies on *Opuntia* and their restriction to more humid coastal areas (Heed 1982).

## Discussion

The sequencing and assembly of the *D. arizonae* and *D. navojoa* genomes resulted in two high-quality draft assemblies with high continuity and completeness. Scaffolds and contigs were ordered based on the reported Muller elements for *D. mojavensis*, giving a better resolution for further studies requiring chromosome location. It is important to notice that in terms of continuity, the *D. arizonae* assembly has 90% of the total bases in just seven fragments, while in *D. navojoa* there are 929 fragments. Neither the *D. navojoa* coding sequence regions nor the subsequent analysis were affected by the higher fragmentation level compared to *D. arizonae*. Nevertheless, after the automated genome improvement (see *Materials and Methods*) the resulting assemblies, based upon their assembly statistics, can be considered high-quality drafts (Chain *et al.*, 2009). In both draft genomes 80% of the total bases localized to the six Muller elements had CEGMA completeness levels of at least ∼84%, considering partial CEG models, confirming their quality.

Detecting evidence of introgression among these species, especially between *D. mojavensis* and *D. arizonae*, is of great interest given that these two species can cross in the laboratory. The fixed inversion differences in three of their six chromosomes complicate the search for introgression because we expect that a hybrid would have a lack of recombination in the inverted regions and regions adjacent to the inversion breakpoints (Wasserman 1962, 1982, 1992; Cirulli and Noor 2007; Machado *et al.* 2007b). Detecting introgression, if it has occurred, would be more likely for the colinear chromosomes. Counterman and Noor (2006) were the first to look for introgression using markers on three chromosomes, finding no evidence of introgression in either inverted or colinear genomic areas. Using 10 markers located across the three large acrocentric inverted and two colinear chromosomes revealed the possibility of past genetic exchange in the colinear chromosomes (Machado *et al.* 2007a). Recent work using short-read data from two strains per species supports that finding (Lohse *et al.* 2015).

We were able to examine rates of single nucleotide substitutions in 9.1 × 10^6^ base pairs of coding sequence distributed among all six chromosomes (three inverted and three colinear). Synonymous and nonsynonymous substitutions were significantly lower in the genes located in colinear chromosomes. Although that finding is consistent with expectations from a secondary contact model in which chromosomal inversions that arise after divergence reduce introgression at some point in the past, we also observed higher divergence levels in two of the inverted chromosomes (Muller A and B) in the pairwise comparisons that include *D. navojoa*, the outgroup species. Thus, although the pattern in Muller E is consistent with the role of inversions in reducing introgression, the interpretation is less clear for Muller A and B. Given that the inversion breakpoints are known for the single inversion Xe in Muller A (Runcie and Noor 2009) and the three overlapping inversions (2q-2r-2s) in Muller E (Guillén and Ruiz 2012), we were able to compare divergences inside and outside the inverted regions for each of those two chromosomes. For Muller E we observed a pattern consistent with the role of the fixed inversion (or rather, the set of overlapping inversions) in reducing introgression between *D. arizonae* and *D. mojavensis* (Table 4). However, for Muller A we did not see any significant differences between colinear and inverted regions in any of the three pairwise species comparisons, but below we propose an explanation based on the relative age of the Xe inversion. The breakpoints of the fixed inversions in Muller B (3a and 3d) (Wasserman 1962, 1992; Ruiz *et al.* 1990) have not been mapped so it was not possible to compare patterns of divergence inside and outside the inverted genomic regions, but we observed that this chromosome shows the largest proportion of genes under positive selection in the outgroup lineage (Figure 4).

In a model assuming contact between species and some gene flow during the divergence process, comparing relative divergences among inverted chromosomes could indicate the relative age of the inversions separating the species (Noor *et al.* 2007). Here, we show that divergences at dS and dN sites for genes located in Muller B were significantly higher than for genes located in Muller E, which in turn were significantly higher than those from Muller A in the comparisons between *D. arizonae* and *D. mojavensis* (Table 3). Barring any differences in mutation rate among inversions, this finding suggests that the set of overlapping inversions located in Muller B arose before those in Muller E, with the inversion in Muller A being the youngest. Given that the Muller A inversion is, potentially, the youngest fixed inversion between *D. arizonae* and *D. mojavensis*, it is possible that enough incompatibilities had already accumulated in that chromosome before the inversion arose. This would not be an unlikely scenario given the well-known large effect of the X chromosome on hybrid incompatibilities (reviewed in Coyne and Orr 2004), and could help explain why the inverted region in this chromosome does not have higher levels of divergence than the colinear region.

How the inversions or their breakpoints relate to the separation of these three species is unclear. A confounding issue is that within each of the three species, structural (Ruiz *et al.* 1990) and nucleotide (Machado *et al.* 2007a) polymorphism exists in the inverted chromosomes. While these three are good species in nature, laboratory crosses utilizing different populations of each species vary greatly in the production of hybrids (Ruiz *et al.* 1990). For example, in crosses between *D. arizonae* females from Guatemala with *D. navojoa* males from Michoacán, 80% of pupae produced adults, while crosses with the same females to male *D. navojoa* from Sonora gave no adults. A relationship between a particular structural variant, let alone a particular nucleotide substitution, and a particular form of incompatibility has never been established. For example, while the colinear chromosomes were implicated in sexual isolation between *D. arizonae* and *D. mojavensis* (Zouros 1982), Reed *et al.* (2007) found that hybrid male sterility was associated with two inverted and one colinear chromosome . Most studies of incompatibilities among these species have focused upon the ability to produce interspecific hybrids in the laboratory.

But their ecological differences provide the opportunity to include the role of host shifts and their relationships to inversions in the divergence among the three species. For example, the ancestral *D. navojoa* is an *Opuntia* breeder. Inversions indicate that *D. arizonae* is somewhat more basal than *D. mojavensis* (Ruiz *et al.* 1990). While *D. arizonae* has acquired the ability to use columnar cacti, it still is widely associated with *Opuntia*, as well as with domestic fruits. Other than the isolated population in Santa Catalina Island, California, *D. mojavensis* breeds exclusively in columnar or barrel cactus. The genetics of these ecological shifts involve processes underlying host localization and utilization, and their relationships to the inversion differences also need to be explored. For example, the restriction of *D. navojoa* to the *Opuntia* species found in western Mexico (Heed 1982) suggests different host adaptations compared to *D. arizonae* and *D. mojavensis*. The more humid habitats in coastal western Mexico and their low desiccation resistance (Matzkin *et al.* 2009), relative to *D. arizonae* and *D. mojavensis*, are evidence of additional genetic differentiation. Further studies should reveal the locations of genes involved in these specializations and their relationship to the inversions and their breakpoints.

Divergence time estimates among *D. repleta* group species have varied, depending upon the type and number of genes utilized. Reed *et al.* (2007), using mtDNA Cytochrome Oxidase I (COI) sequences, estimated that *D. mojavensis* and *D. arizonae* diverged between 0.66 and 0.99 Mya, while Russo *et al.*, (1995) using Adh2, provided a much earlier date of 4.2 Mya (Table S1). In between these dates are other intermediate values based upon Alcohol dehydrogenase (ADH) (Matzkin and Eanes 2003) of 2.4 ± 0.7 and 1.3 Mya, based upon multiple markers across the genome (Lohse *et al.* 2015). Our estimate of 1.51 Mya is based upon whole genome data, but one should keep in mind that the *D. mojavensis* strain sequenced represents only one of the subspecies, and that the value could differ slightly depending upon the subspecies used. With respect to *D. navojoa*, fewer time divergence estimates are available. Machado *et al.* (2007a) inferred, using data from Russo *et al.* (1995), that the split of this lineage occurred 7.8 Mya, while Reed *et al.* (2007) estimated this divergence to have occurred 2.91–4.38 Mya based on mt-COI sequence data, very similar to Matzkin and Eanes’ (2003) estimate using ADH (2.9–4.5 Mya). The divergence time estimated here, 5.85 Mya, falls within the range of previously estimated divergence times and is based on the largest number of sequences so far analyzed.

The complete genomes for *D. arizonae and D. navojoa* should facilitate resequencing the strains of each species that differs in their species-specific chromosomal variants and the association of these structural variants with particular aspects of speciation.

## 

## Supplementary Material

Supplemental Material
