# Supplementary material for: Genome Evolution in Three Species of Cactophilic Drosophila
Source: G3 (Bethesda). 2016 Aug 3;6(10):3097–105. doi: 10.1534/g3.116.033779 (PMC5068933; doi:10.1534/g3.116.033779)
Supplement: Supplemental Material [file supp_6_10_3097__index.html]

Genome Evolution in Three Species of Cactophilic Drosophila — Supplemental Material 

# Genome Evolution in Three Species of Cactophilic *Drosophila*

## Supplemental Material for Sanchez-Flores, *et al*, 2016

**Files in this Data Supplement:**

- Figure S1 - Maximum Likelihood tree of the 14 *Drosophila* species. (.pdf, 43 KB)
- Table S1 - Average synonymous (dS) and nonsynonymous (dN) substitutions per site and dN/dS ratios for coding genes in the inverted and collinear Muller elements in *Drosophila arizonae* (ar), *D. mojavensis* (mo), and *D. navojoa* (na).
- Table S2 - Divergence dates (Ma) for *Drosophila arizonae*, *D. navojoa* and *D. mojavensis*. Comparisons between present and previous studies for *Drosophila* species. (.pdf, 141 KB)
- Table S4 - Number of protein coding genes used in the dN, dS comparisons for each Muller element. (.pdf, 85 KB)
- Table S3 - GO enrichment analyses. (.xlsx, 24 KB)
