# Supplementary figures and images for: Genome Evolution in Three Species of Cactophilic Drosophila
Source: G3 (Bethesda). 2016 Aug 3;6(10):3097–105. doi: 10.1534/g3.116.033779 (PMC5068933; doi:10.1534/g3.116.033779)

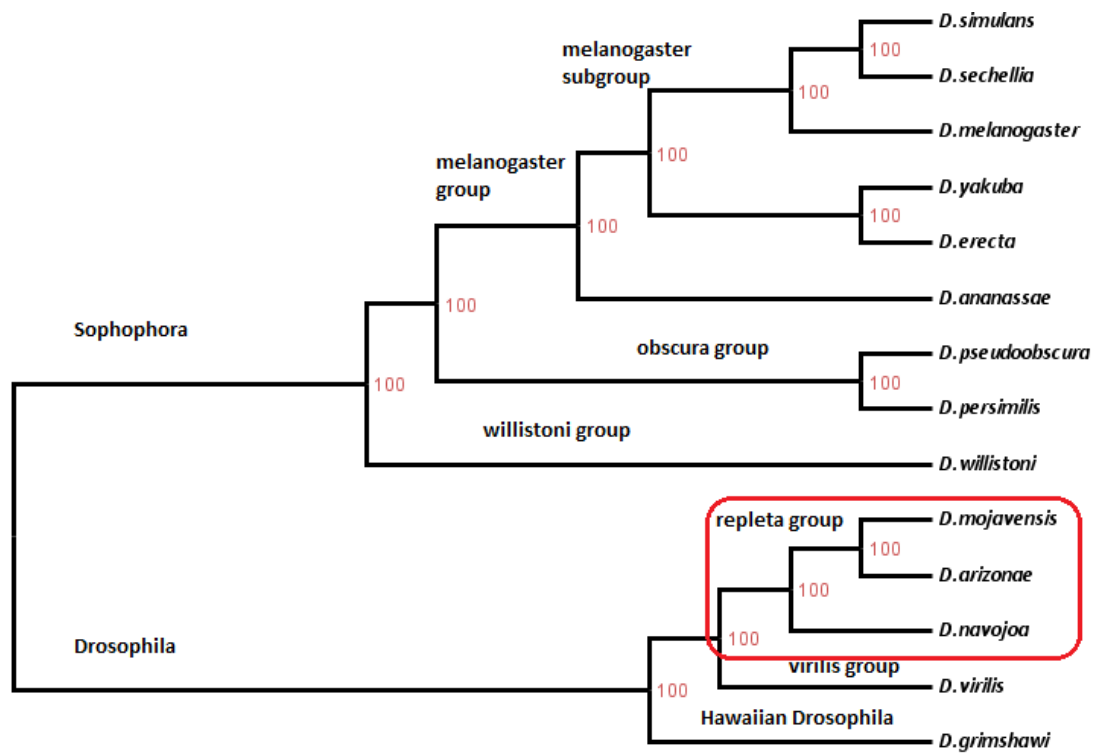

2.0

Supplement: Supplemental Material [file supp_g3.116.033779_FigureS1.pdf]
