# Supplementary material for: Genome Evolution in Three Species of Cactophilic Drosophila
Source: G3 (Bethesda). 2016 Aug 3;6(10):3097–105. doi: 10.1534/g3.116.033779 (PMC5068933; doi:10.1534/g3.116.033779)
Supplement: Supplemental Material [file supp_g3.116.033779_TableS1.pdf]

Table S1. Average synonymous (dS) and nonsynonymous (dN) substitutions per site and dN/dS ratios for coding genes in the inverted and collinear Muller elements in *Drosophila arizonae* (ar), *D. mojavensis* (mo), and *D. navojoa* (na).

	**dN**			**dS**			**dN/dS**		
**Element**	**ar-mo**	**ar-na**	**mo-na**	**ar-mo**	**ar-na**	**mo-na**	**ar-mo**	**ar-na**	**mo-na**
**Inverted**	**0.011**↑	**0.028**↑	**0.028**↑	**0.066**↑	**0.166**↑	**0.167**↑	**0.156**	**0.145**	**0.146**
**Colinear**	**0.008**	**0.022**	**0.023**	**0.056**	**0.151**	**0.150**	**0.161**	**0.139**	**0.143**

↑ Inverted chromosomes with higher divergence levels than collinear chromosomes (Mann-Whitney test; $p<0.05$).
