# Supplementary material for: Genome Evolution in Three Species of Cactophilic Drosophila
Source: G3 (Bethesda). 2016 Aug 3;6(10):3097–105. doi: 10.1534/g3.116.033779 (PMC5068933; doi:10.1534/g3.116.033779)
Supplement: Supplemental Material [file supp_g3.116.033779_TableS2.pdf]

Table S2. Divergence dates (Ma) for *Drosophila arizonae*, *D. navojoa* and *D. mojavensis*. Comparisons between present and previous studies for *Drosophila* species. Ranges in parentheses.

**Nodes**	**Russo *et al*[a]**	**Matzkin *et al*[a]**	**Obbard *et al*[b]**	**Present study**
**Dvir-Dnav**	32 (26 - 38)	-------------	47 (21- 79)	31.13 (25 - 37)
**Dnav-Dmoj**	-------------	3.8 (3.2 - 4.4)	-------------	5.86 (4 - 7)
**Dmoj-Dari**	4.2 (2.22 - 6.18)	2.4 (1 - 3.8)	-------------	1.51 (1.1 – 1.9)

[a] Estimates based on Adh gene.
[b] Estimate based on Hawaiian *Drosophila* calibrations.
