# Supplementary material for: Genome Evolution in Three Species of Cactophilic Drosophila
Source: G3 (Bethesda). 2016 Aug 3;6(10):3097–105. doi: 10.1534/g3.116.033779 (PMC5068933; doi:10.1534/g3.116.033779)
Supplement: Supplemental Material [file supp_g3.116.033779_TableS4.pdf]

Table S4. Number of protein coding genes used in the dN, dS comparisons for each Muller element.

	Darizonae-Dmojavensis	Darizonae-Dnavojoa	Dmojavensis-Dnavojoa
MullerA	1086	1230	1228
MullerB	416	478	478
MullerE	1238	1348	1365
Total inverted	2740	3056	3071
MullerC	1025	1166	1167
MullerD	1057	1211	1216
Total collinear	2082	2377	2383
